# Revealing the Earth’s mantle from the tallest mountains using the Jinping Neutrino Experiment

**DOI:** 10.1038/srep33034

**Published:** 2016-09-09

**Authors:** Ondřej Šrámek, Bedřich Roskovec, Scott A. Wipperfurth, Yufei Xi, William F. McDonough

**Affiliations:** 1 Department of Geophysics, Faculty of Mathematics and Physics, Charles University in Prague, V Holešovičkách 2, 180 00 Praha 8, Czech Republic; 2 Institute of Particle and Nuclear Physics, Faculty of Mathematics and Physics, Charles University in Prague, V Holešovičkách 2, 180 00 Praha 8, Czech Republic; 3 Department of Geology, University of Maryland, College Park, MD 20742, United States; 4 Institute of Hydrogeology and Environmental Geology, Chinese Academy of Geological Sciences, Shijiazhuang, China

## Abstract

The Earth’s engine is driven by unknown proportions of primordial energy and heat produced in radioactive decay. Unfortunately, competing models of Earth’s composition reveal an order of magnitude uncertainty in the amount of radiogenic power driving mantle dynamics. Recent measurements of the Earth’s flux of geoneutrinos, electron antineutrinos from terrestrial natural radioactivity, reveal the amount of uranium and thorium in the Earth and set limits on the residual proportion of primordial energy. Comparison of the flux measured at large underground neutrino experiments with geologically informed predictions of geoneutrino emission from the crust provide the critical test needed to define the mantle’s radiogenic power. Measurement at an oceanic location, distant from nuclear reactors and continental crust, would best reveal the mantle flux, however, no such experiment is anticipated. We predict the geoneutrino flux at the site of the Jinping Neutrino Experiment (Sichuan, China). Within 8 years, the combination of existing data and measurements from soon to come experiments, including Jinping, will exclude end-member models at the 1σ level, define the mantle’s radiogenic contribution to the surface heat loss, set limits on the composition of the silicate Earth, and provide significant parameter bounds for models defining the mode of mantle convection.

Recent cosmochemical observations have produced a range of compositional models for the silicate Earth and its prediction for the amount of radiogenic power in the Earth^1^,^2^,^3^,^4^,^5^. Likewise, new insights on the thermal and electrical conductivity of the Earth’s core^6^,^7^,^8^,^9^,^10^,^11^ have greatly revised our understanding of the core–mantle boundary heat flux, which in turn has significant implications on the nature of the Earth’s surface heat flux. These findings permit a broad range of estimates of the radiogenic power available in the silicate Earth. Of the 46 TW of heat output from the Earth’s interior^12^,^13^, anywhere between ~10 TW and ~30 TW are attributed to the decay of long-lived radionuclides (i.e., ^40^K, ^232^Th, and ^238^U) within existing compositional models^14^. The continental lithosphere accounts for 8 TW^15^ leaving negligible (2 TW; i.e., 10 TW–8 TW) to significant (22 TW) amounts of radiogenic power contributing to mantle dynamics^16^,^17^,^18^,^19^,^20^. The complex and inaccessible deep Earth system, where mantle dynamics is coupled to processes in the metallic core, has so far resisted efforts to better constrain the K, Th, U abundance in the Earth.

Compositional models of the Earth have been categorized into three groups based on the available radiogenic power^21^,^22^: low-Q models (10–15 TW), medium-Q models (17–22 TW), and high-Q models (>25 TW). Low-Q models assume a low K, Th, and U concentration in the material that formed the Earth (the enstatite chondrite model and the non-chondritic model) or invoke an impact-induced loss of early differentiated crust enriched in heat-producing elements (the collisional erosion model). Medium-Q models estimate the silicate Earth composition using elemental fractionation patterns between melt (basalt) and melt residue (peridotite) while constraining the ratios of refractory lithophile elements to abundances in C1 chondritic meteorites. High-Q estimates are the high end-member of physical models which rely on simple relationship between the heat output from the convecting mantle and the vigor of convection, described as a balance between thermal buoyancy driving the dynamics and thermal and momentum diffusion hindering the flow.

The recent breakthrough in detection of terrestrial electron antineutrinos, created in *β*^−^ decays of ^232^Th and ^238^U decay chains of natural thorium and uranium, has offered an exciting new framework for studying the shallow and the deep Earth’s composition and for tightening constraints on the amount of radiogenic heat available for driving Earth’s dynamics. It took 26 years from Wolfgang Pauli’s original proposal of a neutrino in 1930 to the first detection of antineutinos by Reines and Cowan in 1956^23^. An additional almost 50 years passed before the first detection of geoneutrinos with the KamLAND 1-kiloton liquid scintillator detector at Kamioka Underground Laboratory in Japan in 2005^24^. A few years later the Borexino collaboration released their initial measurement of the Earth’s geoneutrino flux with the 0.3-kton detector at Gran Sasso (Italy)^25^.

These two neutrino experiments will be soon joined by the 1-kton SNO+ detector at SNOLAB (Ontario, Canada)^26^ and a fourth experiment, the 20-kton JUNO detector, which is under construction in Jiangmen (China)^27^. In addition, a prototype detector is currently being built at the China Jinping Laboratory (CJPL; Fig. 1). Following this testing phase the Jinping Neutrino Experiment^28^ (hereafter Jinping) is designed to build a 4-kton detector for low-energy neutrino physics, astrophysics and geophysics at the CJPL. Importantly, CJPL is the world’s deepest underground physics laboratory where a rock overburden of ~2400 m (6700 meters water equivalent)^28^ results in the lowest flux of cosmic ray muons, thus minimizing the unwanted cosmogenic background in antineutrino detection. Furthermore, CJPL is remote from nuclear reactors which also emit electron antineutrinos, with the nearest operating reactor 1400 km away. Jinping will thus give an unprecedented antineutrino measurement dominated by the geoneutrino signal^28^, unlike any other geoneutrino detecting experiment^29^.

KamLAND (KL) and Borexino (BX) geoneutrino measurements^24^,^25^,^30^,^31^,^32^,^33^ are broadly consistent with existing models of Earth’s architecture and its chemical composition, thus independently validating the geoscientific paradigms—i.e., Bulk Silicate Earth (BSE) Th and U abundance estimates, and enrichment of the heat-producing elements in the crust. These experiments have also demonstrated that the existing budget of heat producing elements is insufficient to account for the 46 TW of surface heat flow^30^, thus requiring the presence of residual primordial energy, which includes the heat of accretion and the transformation of gravitation energy of core formation into thermal energy. Furthermore, an upper limit has been placed on thermal power of a nuclear geo-reactor at depth^31^, proposed by some^34^,^35^. Geoneutrino research is now entering the exciting next stage where geoneutrino measurements begin to address the large uncertainty in estimates of radiogenic power driving mantle convection, stemming from various models of Earth’s composition. Most recently the signal of geoneutrinos from the mantle has been reported^33^,^36^,^37^, although with a considerable uncertainty.

In this report, we calculate the prediction of the geoneutrino flux at Jinping. We demonstrate the power which the Jinping measurement will bring in combination with results of the earlier geoneutrino experiments. Moreover, we make a case for the critical role of constructing an accurate crustal emission model from nearby crust at Jinping, in resolving the mantle signal.

## Emission model and results

Our global model for geoneutrino emission (see Methods section) integrates the three-dimensional spatial structure and rock density from CRUST1.0^38^ (C1) with estimates of chemical composition in various reservoirs: layers of Continental Crust (CC) and Oceanic Crust (OC) including sediment layers, Continental Lithospheric Mantle (CLM), and the convecting mantle composed of the Depleted MORB-source Mantle (DM; source for mid-oceanic ridge basalts), and the Enriched Mantle (EM). The EM is introduced in order to satisfy the mass balance of elements in the Bulk Silicate Earth (BSE) and is a source of oceanic intraplate basalts (OIB). We do not make a distinction between several types of enriched chemical reservoirs in the deep Earth as seen by geochemistry (e.g., the EM1, EM2, and HIMU reservoirs^39^), as such compositional differences will remain beyond detection sensitivity^14^. Various compositional estimates result in a suite of models whose calculated antineutrino emission can be tested with geoneutrino measurements. Here we calculate the geoneutrino predictions for a typical Earth model^15^ as a reference, whereas the Supplementary information reviews the consideration for the complete spectrum of competing Earth models.

Table 1 lists geoneutrino fluxes at the Jinping location, 28.15°N, 101.71°E, that come from the distinct geochemical reservoirs of the Earth model (Supplementary Figure S1). Uncertainty in the predicted flux are dominated by unknowns in the chemical composition of the layers, whereas uncertainties in crustal thickness are uncorrelated and estimated to be <10%, while not reported in C1. Accounting for the uncertainty in crustal structure is expected to increase the uncertainty in lithospheric geoneutrino flux prediction by a few percent, resulting in a larger relative uncertainty of the mantle flux, given the ratio of lithospheric to mantle flux at Jinping and other continental locations of neutrino experiment. The total predicted geoneutrino signal at Jinping is 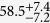 TNU (Terrestrial Neutrino Units^40^), with 86% of the signal from the lithosphere (crust + CLM) and 14% from the convecting mantle (DM + EM).

## Resolving mantle

Determining the amount of radiogenic heat production in Earth’s mantle is a major goal in the field. Such a constraint will transform our understanding of the composition of the silicate Earth, mantle dynamics and the cooling history of the planet. To be able to unambiguously define the mantle-only geoneutrino signal means deploying a detector deep in the oceans (or buried on an ocean island) far away from nuclear reactors and continental lithosphere. Both the reactor antineutrino background and the lithospheric signal prediction must be subtracted from the total antineutrino measurement and reducing these contributions increases the relative proportion of mantle signal while reducing uncertainty. Such an ocean-going experiment has been proposed, i.e., Hanohano^41^. However, it may take decades before Hanohano or a similar experiment is approved and operational. In the absence of a detector located in the middle of the ocean, Jinping is our best solution as it will provide critical data in defining the mantle contribution.

The power of the Jinping experiment comes from the potential of a precise geoneutrino detection, given developments in the field in the last decade and the specifics of its location. Jinping will detect the largest geoneutrino flux (TNU signal) of all geoneutrino detectors (Fig. 2). Because of low cosmogenic and reactor antineutrino background, Jinping is expected to measure geoneutrinos with the greatest precision of all detectors, quantified as relative uncertainty of 4% after an exposure of a 3-kiloton target mass over 5 years^28^. The limiting factor of resolving the mantle geoneutrino flux using Jinping measurement is the uncertainty in the prediction of the lithospheric signal, which must be subtracted from the total measurement. In our geoneutrino emission model the uncertainty in the lithospheric flux simply scales with the lithospheric flux magnitude and is therefore comparatively large at Jinping.

**Figure 1:**
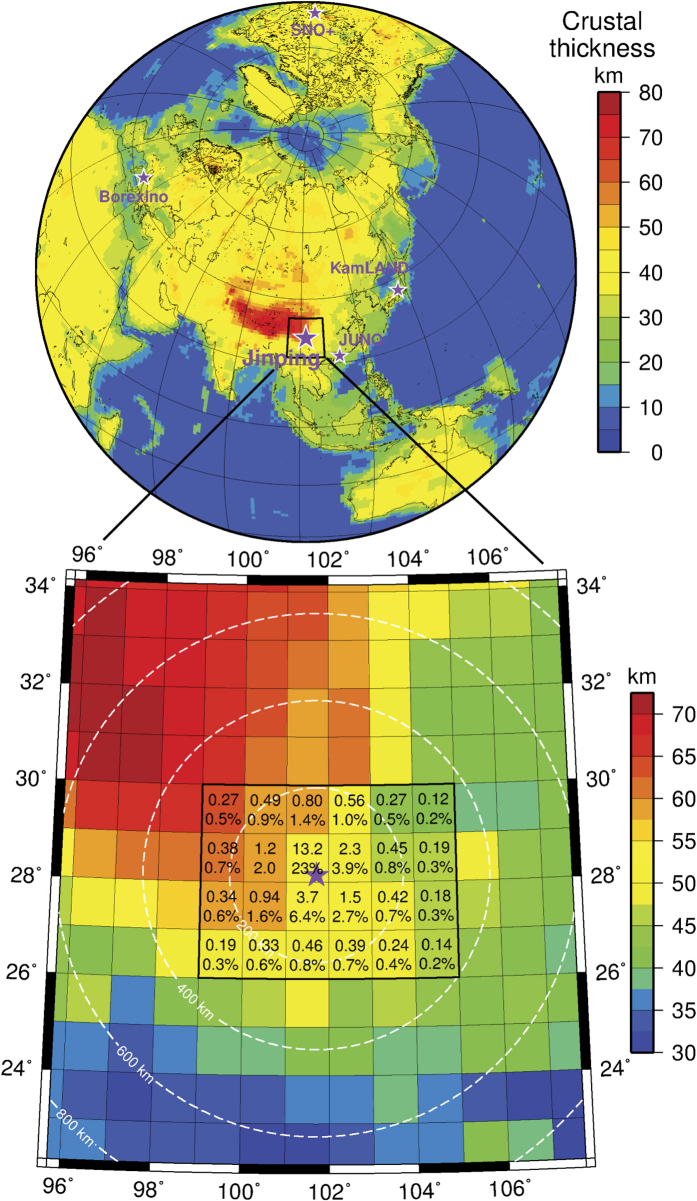
**Top**: Location of Jinping and other geoneutrino detectors. Crustal thickness from CRUST1.0^38^ model plotted in color. **Bottom:** 1°longitude × 1°latitude tiles of CRUST1.0 model around Jinping. Within the 6° × 4° region centered at the detector location (somewhat arbitrarily defined and termed “near-field crust” in past studies) we show TNU (Terrestrial Neutrino Units^40^) and % contributions from the lithosphere (i.e., Continental Crust + Continental Lithospheric Mantle) in each tile to the total geoneutrino signal at Jinping. White dashed circles contour distance from Jinping. Map created using The Generic Mapping Tools, Version 4.5.14 (http://gmt.soest.hawaii.edugmt.soest.hawaii.edu).

**Figure 2:**
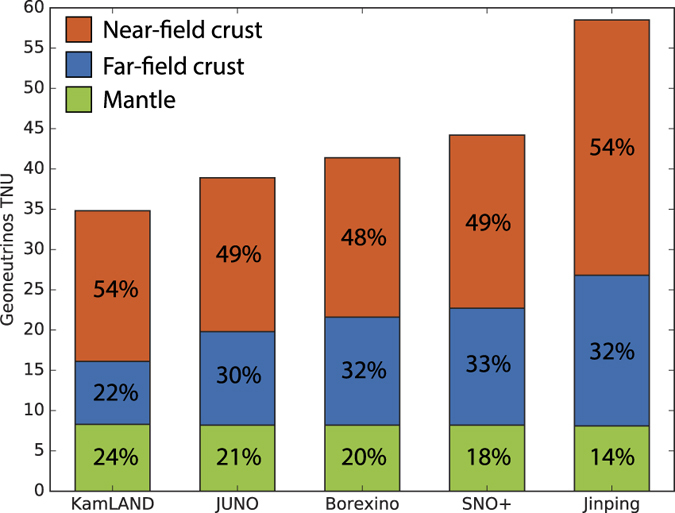
Geoneutrino flux predictions at geoneutrino detectors, showing contributions from Near-field crust (NFC), Far-field crust (FFC), and the convecting Mantle (DM + EM). NFC is a 6°longitude by 4°latitude region centered at the detector location. NFC and FFC include the small contribution (<2 TNU) from the underlying Continental Lithospheric Mantle (CLM). See Fig. 1 for detector locations and TNU.

**Figure 3:**
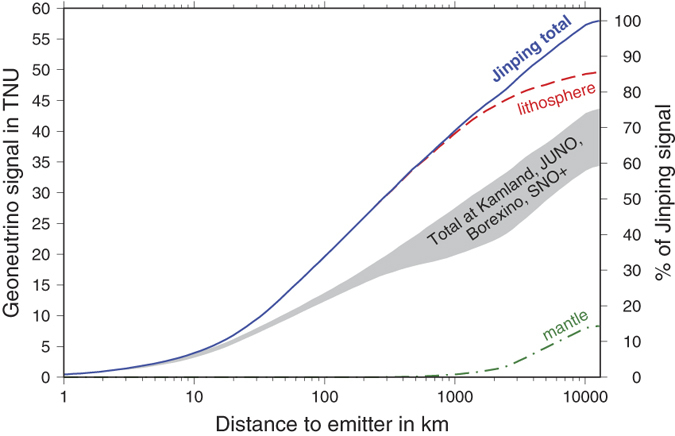
Cumulative geoneutrino signal vs. distance to emission location at Jinping. Showing both TNU (left vertical axis) and % of total signal (right axis). Total signal and contributions from lithosphere (crust + CLM) and mantle (DM + EM) are plotted. Grey shaded area envelops signals at detectors KamLAND (lower bound), JUNO, Borexino, and SNO + (upper bound).

It has been recognized that a large fraction of the expected geoneutrino flux at a detector originates from the closest few hundred km surrounding a detector^24^. Figure 1 shows the lithospheric contribution to the geoneutrino flux coming from the surrounding 1°longitude × 1°latitude tiles of the C1 discretization. Almost a quarter of the signal (23%) originates in the tile in which Jinping sits. The plot of cumulative geoneutrino flux versus distance to emitter (Fig. 3) at Jinping shows the steepest sloping curve of all detectors, where 50% of signal originates within 300 km distance, 60% within 500 km, and 70% within 1000 km. Thus, it is fundamentally important to characterize the local geology as it represents the largest contributor to the signal and uncertainty on the total expected flux. The geoneutrino flux estimates from the local lithosphere must become constrained by multiple geophysical and geochemical observables including existing heat flow data, seismic observations, gravity data, and measured element abundances in rocks. Local crustal studies have been performed around KamLAND, Borexino, and SNO+ and constitute an urgent challenge for geoscience in geoneutrino research at Jinping and JUNO.

The area around Jinping has been heavily studied because of the many devastating earthquakes that have occurred in the region, with the most recent ones being the 2008 Wenchuan (Sichuan) earthquake and the 2013 Lushan earthquake^42^,^43^,^44^,^45^,^46^. Furthermore, Jinping is sited on the eastward facing ramp of the Tibetan Plateau that abuts the Sichuan Basin and is known to be located in one of the world’s fastest moving geological regions, with vertical uplift rates reaching up to 6 mm/yr and horizontal movements exceeding 10 mm/yr^47^. Hundreds of GPS measurements and identification of the many major tectonic faults reveal large scale tectonic block rotation and crustal flow in the region^48^,^49^,^50^,^51^. This region has been and continues to be intensely studied for both understanding the fundamental processes of plate tectonics and to improve our abilities to predict the occurrence and consequences of major earthquakes.

**Figure 4:**
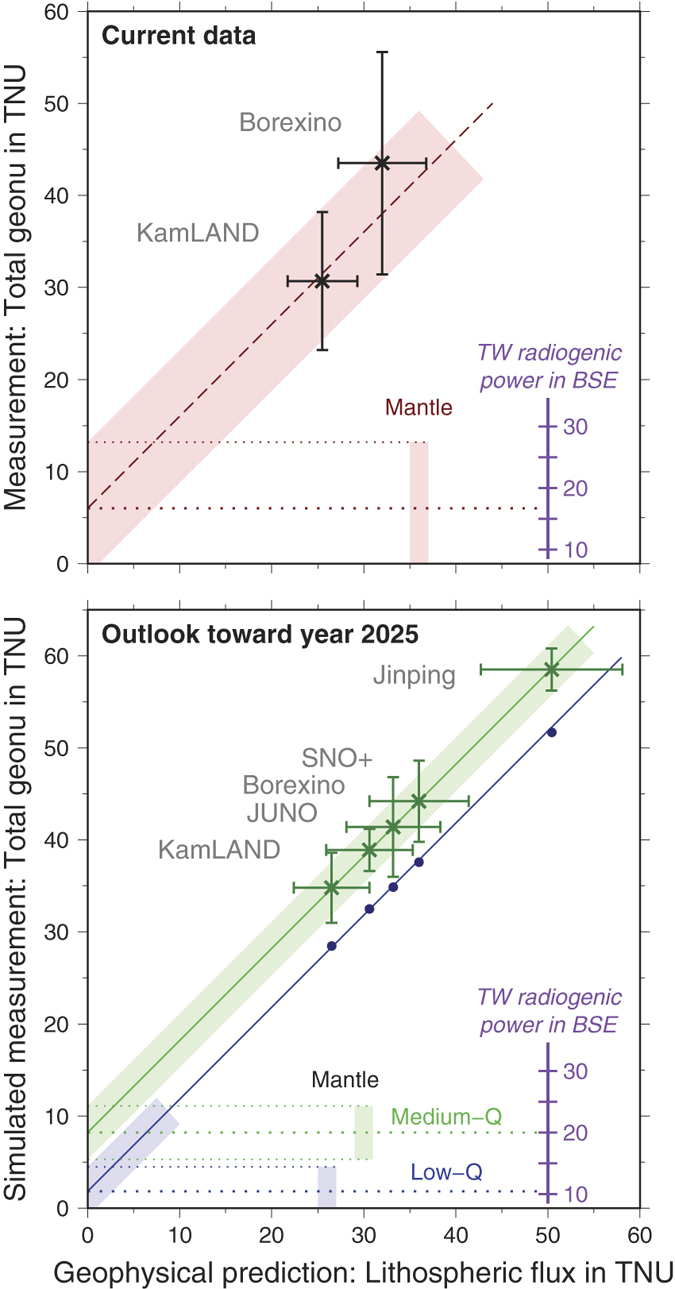
**Top**: Most recent measurement of total geoneutrino flux at KamLAND (KL)^31^ and Borexino (BX)^33^ (vertical axis) vs. lithospheric flux prediction (this study). Best fit of slope 1 line shown as red dashed line, including ±1*σ* uncertainty (red band). The y-intercept reveals signal from the convecting mantle (DM + EM), which scales with radiogenic power in BSE (purple). **Bottom:** Simulated measurements in year 2025 (vertical axis) vs. lithospheric predictions at geoneutrino detectors KL, JUNO, BX, SNO+, and Jinping (JP). Assumes that detectors measure the nominal value predicted by the emission model, and measurement uncertainty is assumed to be 11% (KL)^52^, 6% (JUNO)^53^, 13% (BX), 9% (SNO+), and 4% (JP)^28^, respectively. We show results for two BSE compositional estimates, previously termed medium-Q and low-Q models^21^,^58^. The solution of mantle flux for the medium-Q model translates into 12 ± 4 TW of radiogenic power in the mantle.

Even though the mantle signal at Jinping is a small fraction (14%) of the total geoneutrino signal, the power of combining the Jinping measurement with other experiments is unprecedented. Figure 4 illustrates this feature with a plot that compares the measured geoneutrino flux (ordinate, physics only input) versus the geological estimate of the flux from the lithosphere (i.e., crust + CLM; abscissa), with the flux from convecting mantle (DM + EM) being the remaining contribution. Consequently, fitting the data with a line of slope 1 yields the y-intercept, which identifies the mantle contribution to the total signal, and provides its uncertainty as a function of the unknowns in the geoneutrino measurements (i.e., the experimental neutrino physics uncertainty) and in the lithospheric flux predictions (i.e., the uncertainty in geological model). This analysis can be repeated for each experiment individually or any combination of experiments. Analyses on the existing data (KL and BX combined) provides a result with a large uncertainty on the mantle flux (i.e., 6.0 ± 7.2 TNU for the y-intercept; Fig. 4, top; see Supplementary information for details). By the time Jinping produces a measurement, other detectors will have accumulated additional data.

The existing geoneutrino experiments are statistics limited, so with more exposure the relative uncertainties in their signal drop as the inverse square root of the measurement following Poisson’s statistics. The annual geoneutrino count rate is predicted to be about 400 at JUNO, 100 at Jinping, 20 at SNO+, and it has been measured as 14 at KamLAND and 4.2 at Borexino. Given the marked reduction in the reactor signal following the Tohoku 2011 earthquake, KamLAND is on track to reach 11% uncertainty in 7 more years of counting^52^. The 20-kton JUNO detector will provide a significant annual flux of geoneutrino events and improvements in characterizing and subtracting the reactor signal (estimated at 3% uncertainty) will yield a geoneutrino measurement with 6% uncertainty after 5 years of live time^53^. Extrapolating the statistics of current Borexino measurements^25^,^32^,^33^, we predict an uncertainty of 13% after 6 additional years. SNO+ detector’s assumed count rate of 20 geoneutrinos per year gives an estimate of 9% measurement uncertainty after 6 years^26^,^54^. A projection for the year 2025, based on all of the detectors expected to be online (KL, BX, SNO+, JUNO, and Jinping), reduces the uncertainty of the result of mantle flux, 8.2 ± 2.9 TNU, down to 35% relative uncertainty for the tested model (Fig. 4, bottom). With this reduction in uncertainty on the mantle flux, by a factor of 2.5 relative to the current result using KL and BX data, we will clearly discriminate between models of silicate Earth composition and put narrow bounds on radiogenic power in the mantle. It is also seen in Fig. 4 that while the measurement uncertainty at Jinping is the smallest, the uncertainty in the lithospheric flux prediction is the largest of all detectors, as in the present model it simply scales with the flux magnitude. Its reduction offers the greatest potential to further pin down the mantle contribution.

The Jinping detector and Fig. 4 offers critical insights into the nature of geoneutrino science. Each of these five detectors can independently see the mantle given the slope 1 requirement. Differences in the intercept value reflect one of three potential considerations: (1) biases in the detectors, (2) variations in the mantle flux, and/or (3) biases in the predicted crustal flux. Assuming that instrumental calibrations reduce detector bias and total variation in mantle fluxes is expected to be at the 10% level^14^, then deviations in the y-intercepts can be in turn used to interrogate the assumed crustal model for the detector. Coupling data from continental based detectors with constraints from an oceanic based detector will provide unprecedented opportunities to critically evaluate competing models of crust composition. In this regard Jinping represents a significant test case with its exceptionally thick crust and distinctly bright geoneutrino flux.

**Figure 5:**
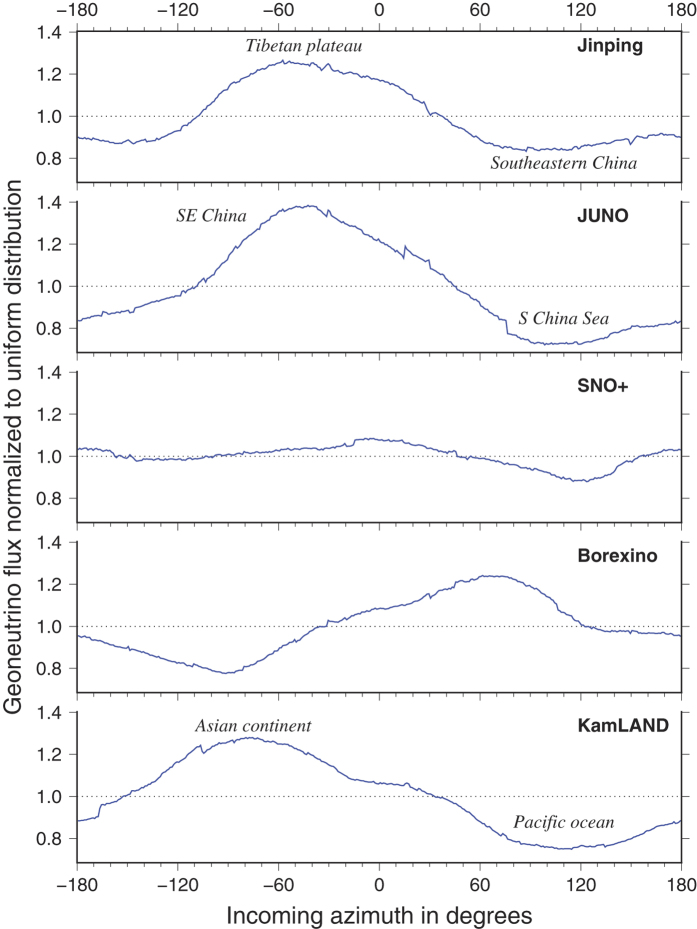
Predicted signal at geoneutrino detectors as a function of azimuth of incoming geoneutrino. Normalized to uniform distribution at each detector.

Recent advances in antineutrino detection technology have been in directionality studies^55^. Being able to evaluate directionality, even at 180° resolution, provides a powerful documentation of the sources of the geoneutrino signal (i.e., distinguishing near field crustal contributions that can be up to 50% of the signal). Primary focus in geoneutrino directionality analyses has been the variation of the crust and mantle signals with the incoming dip angle^56^,^57^. In Fig. 5 we predict the normalized azimuthal distribution of the geoneutrino signal at the various detectors. The asymmetric azimuthal signal at KamLAND, Borexino, and JUNO detectors reflects their settings on the margins of continents. The least variable azimuthal signal is seen for SNO+, which sits in the center of the North American plate. The asymmetry in Jinping’s azimuthal signal reflects the exceptionally thick continental crust of the Himalayas to the west and the normal ~40 km crust of eastern China. While currently unable to measure geoneutrino directionality, predictions of azimuthal signal intensity provide insight into the geology of the local crust and inform mapping and sampling efforts for regional geologic models.

## Conclusion

The predicted geoneutrino signal for the proposed Jinping Neutrino Experiment is 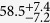 TNU, of which 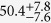 TNU is from the Crust + Continental Lithospheric Mantle and 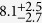 TNU is from the Depleted + Enriched Mantle. The Jinping measurement, combined with geoneutrino measurements at other continental sites, is currently our best chance at resolving the mantle signal. Dedicated geophysical effort toward an accurate local lithospheric model is required. This is a realistic goal, given the wealth of geophysical data in this well studied seismogenic region at the boundary between the Tibetan Plateau and the Sichuan Basin.

Refinement to model predictions of the lithospheric flux are crucial to reducing the uncertainty estimates of the mantle flux. The strategy mapped out here reveals that geoneutrino data will constrain the amount of radiogenic heat production in the mantle by combining all measurements from continental detection sites to reduce the uncertainty. Reference model predicts that constraining the mantle’s radiogenic heat production to 12 ± 4 TW is achievable within 8 years. Such a strategy will successfully discriminate between models of the Earth’s composition, i.e., the previously described low-Q, medium-Q, and high-Q models predicting anywhere from 2 TW to >20 TW of radiogenic power in the mantle^14^,^21^,^22^. These data will place limits on the amount of heat producing elements inside the Earth, describe the planetary abundances of the refractory lithophile elements, and thus define the building blocks of the Earth^58^. Moreover, by setting a limit on the radiogenic heat production in the mantle we will constrain the Urey ratio of the convecting mantle (Ur = radiogenic heat/total mantle heat flux), a parameter that is considerably debated (i.e., estimates of Ur from 0.2 to 0.7) in the literature^16^,^17^,^18^,^19^,^20^.

## Methods

The geoneutrino flux at Jinping location is calculated in the usual way^37^,^40^,^59^,^60^. Flux *ϕ* at location 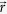 is the integral


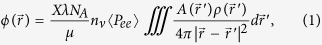


where meanings of various quantities are described in Table 2. As we assume negligible Th, U in the core^61^, the integration domain is the Earth’s crust and mantle, where antineutrino emitters reside. We average the effect of neutrino oscillations by using the average survival probability 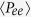. We use CRUST1.0 model^38^ (C1) to describe the geometry and rock density in the crust. C1 parametrizes the crust as 1° latitude by 1° longitude stacks of 6 tiles (excluding ice and water layers) of a given thickness and uniform density. Depth-dependent density in the mantle is taken from PREM^62^. We divide the crust into Oceanic Crust (OC; ‘A’ and ‘B’ type tiles of C1) and Continental Crust (CC). Continental Crust is underlain by the Continental Lithospheric Mantle (CLM), which is assumed to extend to 175 km depth^15^. The bulk of the mantle is divided into two reservoirs, the Depleted Mantle (DM) and the Enriched Mantle (EM) where EM is a layer of uniform thickness at the base of the mantle containing 18% of mantle mass^63^ (i.e., layering at radius of 4200 km). Within each of the chemical reservoirs (i.e., layers of the crust in CC and OC, CLM, DM, EM), the abundance of Th, U is assumed uniform, with values and their uncertainties adopted from several compositional estimates (see Table 3). Abundances in EM are calculated to balance the overall inventory in BSE. A sketch of the model, showing the global structure and the distinct chemical reservoirs, is shown in Supplementary Figure S1.

Uncertainty on the structure and rock density is not available within CRUST1.0, and is not considered in the emission model. Uncertainty in the input abundances of Th and U is propagated using a Monte Carlo approach. The selection of CLM abundances is assumed to follow a log-normal distribution^15^. Abundances in other reservoirs (layers of CC and OC, DM, BSE) are assumed to follow the normal distribution^64^. We assume that Th and U abundances within a reservoir are fully correlated when performing their Monte Carlo fluctuations. We further assume that abundances are uncorrelated between the following reservoirs: BSE, CLM, layers of CC and OC (Supplementary Figure S1). We find, however, that some degree of correlation must be introduced between abundances in DM and the rest of the model, in order to prevent unphysical situations where abundances in EM are below DM values or even negative. The somewhat smaller absolute uncertainty in the total predicted geoneutrino flux compared to the lithospheric flux (Table 1) results from the anti-correlation between abundances in EM and abundances in layers of the lithosphere and in DM when balancing the inventory of elements in BSE.

## Additional Information

**How to cite this article**: Šrámek, O. *et al*. Revealing the Earth’s mantle from the tallest mountains using the Jinping Neutrino Experiment. *Sci. Rep.* **6**, 33034; doi: 10.1038/srep33034 (2016).

## Supplementary Material

Supplementary Information

## Figures and Tables

**Table 1:** Prediction of geoneutrino flux at Jinping location: 28.15°N, 101.71°E, 2400 m depth, based on CRUST1.0^38^ model of the crustal structure.

Reservoir	Geoneutrino flux in TNU†		
	Th	U	Th + U
Upper CC + sediments	7.37 ± 0.74	28.3 ± 6.0	35.7 ± 6.7
Middle CC	2.70 ± 0.22	8.1 ± 2.5	10.8 ± 2.7
Lower CC	0.292 ± 0.088	0.72 ± 0.22	1.02 ± 0.31
OC sediments	0.032 ± 0.002	0.102 ± 0.005	0.134 ± 0.008
OC crust	0.009 ± 0.003	0.045 ± 0.013	0.054 ± 0.016
CC + OC	10.40 ± 0.77	37.3 ± 6.5	47.7 ± 7.2
CLM	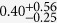	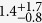	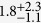
CC + OC + CLM	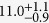	39.3 ± 6.8	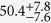
Depleted Mantle (DM)	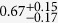	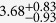	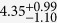
Enriched Mantle* (EM)	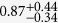	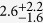	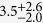
DM + EM	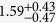	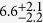	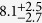
TOTAL	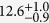	45.9 ± 6.4	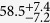

^*^See text for details on how the EM was determined to satisfy BSE model.

^†^See text for details on units. CC = Continental Crust; OC = Oceanic Crust; CLM = Continental Lithospheric Mantle.

**Table 2:** Quantities used is geoneutrino flux calculations.

Quantity	Symbol	^40^K	^232^Th	^235^U	^238^U	Reference
Natural isotopic mole fraction	*X*	0.000117	1	0.007204	0.992742	65
Standard atomic mass (g/mol)	*μ*	39.0983	232.038	238.029	238.029	65
Half-life (10^9 ^yr)	*t*_1/2_	1.248	14.0	0.704	4.468	66
Decay constant (s^−1^)	*λ*					*λ* = ln2/*t*_1/2_
Number of 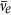 ’s per decay	*n*_*v*_	0.8928	4	4	6	66
Heat per decay (10^−12 ^J)		0.110	6.475	7.108	7.648	67
Elemental abundance (mass fraction)	*A*					see Table 3
Rock density (g/cm^3^)	*ρ*					from CRUST1.0 or from PREM models
Avogadro’s number (mol^−1^)	*N*_*A*_					
Average survival probability	〈*P*_*ee*_〉 = 0.553					67, 68
cm^−2^ *μ*s^−1^ to TNU conversion factors	^232^Th:	0.25 cm^−2^ *μ*s^−1^ TNU^−1^				67
	^238^U:	0.076 cm^−2^ *μ*s^−1^ TNU^−1^				67

**Table 3:** Abundance estimates (in kg/kg) used as inputs in the geoneutrino emission model.

	K	Th	U	Ref.
Upper CC + sediments	(2.32 ± 8%) × 10^−2^	(10.5 ± 10%) × 10^−6^	(2.7 ± 21%) × 10^−6^	64
Middle CC	(1.91 ± 14%) × 10^−2^	(6.5 ± 8%) × 10^−6^	(1.3 ± 31%) × 10^−6^	64
Lower CC	(0.51 ± 30%) × 10^−2^	(1.2 ± 30%) × 10^−6^	(0.2 ± 30%) × 10^−6^	64
OC sediments	(1.83 ± 7%) × 10^−2^	(8.10 ± 7%) × 10^−6^	(1.73 ± 5%) × 10^−6^	69
OC crust	(716 ± 30%) × 10^−6^	(0.21 ± 30%) × 10^−6^	(0.07 ± 30%) × 10^−6^	70
CLM	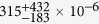	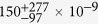	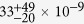	15
Depleted Mantle	(152 ± 20%) × 10^−6^	(21.9 ± 20%) × 10^−9^	(8.0 ± 20%) × 10^−9^	71
Enriched Mantle*	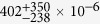	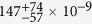	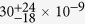	
Bulk Silicate Earth	(280 ± 21%) × 10^−6^	(80 ± 15%) × 10^−9^	(20 ± 20%) × 10^−9^	72

^*^Abundance in Enriched Mantle calculated from balance of each element (BSE = CC + OC + CLM + DM + EM) where EM is 18% by mass of the convecting mantle^63^.
