# Supplementary material for: Revealing the Earth’s mantle from the tallest mountains using the Jinping Neutrino Experiment
Source: Sci Rep. 2016 Sep 9;6:33034. doi: 10.1038/srep33034 (PMC5017162; doi:10.1038/srep33034)
Supplement: Supplementary Information [file srep33034-s1.pdf]

Supplementary information

Revealing the Earth's mantle from the tallest mountains using the Jinping Neutrino Experiment

Ondřej Šrámek[1,*], Bedřich Roskovec[2], Scott A. Wipperfurth[3], Yufei Xi[4], and William F. McDonough[3]

[1]Department of Geophysics, Faculty of Mathematics and Physics, Charles University in Prague, V Holešovičkách 2, 180 00 Praha 8, Czech Republic
[2]Institute of Particle and Nuclear Physics, Faculty of Mathematics and Physics, Charles University in Prague, V Holešovičkách 2, 180 00 Praha 8, Czech Republic
[3]Department of Geology, University of Maryland, College Park, MD 20742, United States
[4]Institute of Hydrogeology and Environmental Geology, Chinese Academy of Geological Sciences, Shijiazhuang, China
[*]ondrej.sramek@gmail.com

Chemical reservoirs

Figure S1 shows the Earth's chemical reservoirs in the geoneutrino emission model setup, including references to the compositional models. Chemical abundances of K, Th, and U are listed in Table 3 of the paper.

Alternative BSE compositional estimates

Our reference geoneutrino emission model uses Bulk Silicate Earth composition of 280 ± 60 ppm K, 80 ± 13 ppb Th, 20 ± 4 ppb U[9] and generates 20 ± 4 TW of radiogenic power (i.e., a medium-Q model[10–12]), contributing less than half of Earth's surface heat flux. To investigate the full spectrum of proposed Silicate Earth compositions, we follow our previous work[13] and construct two additional models which differ from the reference model by having a smaller or a larger amount of heat-producing elements and the corresponding geoneutrino emission from the mantle. The low-Q model composition is 146 ± 39 ppm K, 43 ± 4 ppb Th, 12 ± 2 ppb U, which outputs 11 ± 2 TW of radiogenic power. The high-Q model composition is 350 ± 35 ppm K, 140 ± 14 ppb Th, 35 ± 4 ppb U, which outputs 33 ± 3 TW of radiogenic power. The prospects of discriminating between the models using combined analysis of geoneutrino measurements are illustrated in Figure 4 of the paper and detailed below.

Mantle result from combining Kamland and Borexino measurements

KamLAND's most recent geoneutrino measurement[14], expressed in TNU, is 30.7 ± 7.5 TNU. Borexino latest results is $43.5^{+12.1}_{-10.7}$ TNU[15]. As we do not know the actual statistics of the results, we assume symmetrical Gaussian uncertainties and use measurement data points 30.7 ± 7.5 TNU (Kamland) and 43.5 ± 12.1 TNU (Borexino). Our geoneutrino emission model predicts the lithospheric flux to be 26.5 ± 4.1 TNU at KamLAND and 33.2 ± 5.1 TNU at Borexino. Combining the current total geoneutrino measurements and lithospheric flux prediction from KamLAND and Borexino yields a mantle result of 6.0 ± 7.2 TNU.

Mantle result from combining simulated measurements at 5 detectors

We use our geoneutrino emission model to construct a simulated dataset for combined analysis at the five detectors expected to measure by 2025: KamLAND, Borexino, SNO+, JUNO, and Jinping. We assume that the detectors measure the total geoneutrino flux predicted with the emission model and the measurement uncertainty is estimated as follows. KamLAND is expected to reach 11 % measurement uncertainty in additional 7 years of counting[16].

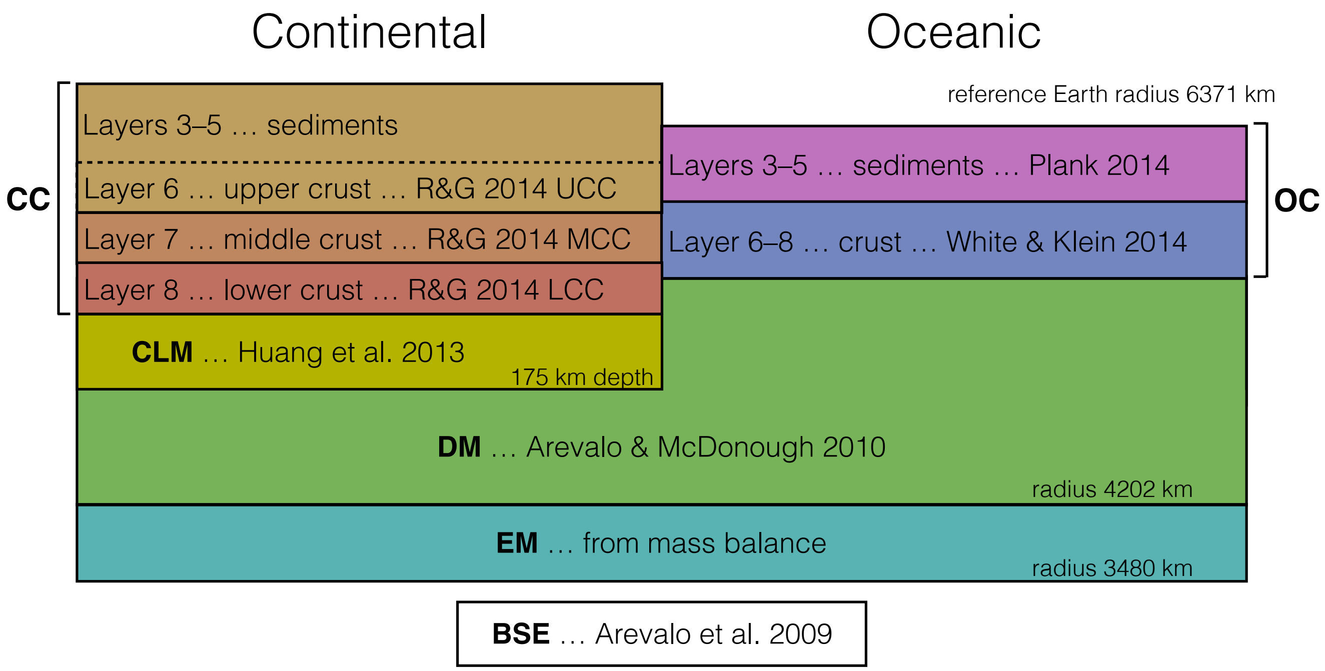

Figure S1. Sketch of layers of the Earth model. CC = Continental Crust, OC = Oceanic Crust, CLM = Continental Lithospheric Mantle, DM = Depleted Mantle, EM = Enriched Mantle. Geometry (i.e., laterally varying thickness of crustal layers) and rock density in the crust are taken from CRUST1.0[1] model. Additionally, Supplementary Table S2 shows result calculated with LITHO1.0[2] model of the lithosphere. Depth-dependent density from PREM[3] in used in CLM, DM, and EM. Different color means different composition, uniform within each reservoir, obtained from estimates in Table 3 of the paper: R&G 2014[4], Plank 2014[5], White & Klein 2014[6], Huang et al. 2013[7], Arevalo & McDonough 2010[8], and Arevalo et al. 2009[9].

Uncertainty of JUNO measurement was estimated at 6 % after 5 years of live time[17]. Jinping is expected to provide a measurement with uncertainty of 4 % after an exposure of a 3-kiloton target mass over 5 years[18]. We estimate the uncertainty of SNO+ result to be 9 %, obtained as the statistical uncertainty of 20 counts per year over 6 years. Similarly for Borexino, we extrapolate the statistics of existing measurements and predict an uncertainty of 13 % after additional 6 years of live time. Using the simulated measurements and lithospheric prediction, we combine the data from all five detectors which yields a mantle result of 8.2 ± 2.9 TNU for the reference model (medium-Q model). Resulting mantle flux for the low-Q model is 1.8 ± 2.7 TNU, and the high-Q model gives 17.1 ± 3.1 TNU.

Table S1. Predictions of total and lithospheric geoneutrino flux and values of the assumed measurement uncertainty at the five detectors.

	Rad. power TW	KamLAND TNU	JUNO TNU	Borexino TNU	SNO+ TNU	Jinping TNU
Lithospheric geoneutrinos	8.2	26.5 ± 4.1	30.6 ± 4.7	33.2 ± 5.1	36.0 ± 5.4	50.4 ± 7.7
Total geoneutrinos, low-Q model	11.4	28.5	32.5	34.9	37.6	51.7
Total geoneutrinos, medium-Q model	20.4	34.8	38.9	41.4	44.2	58.5
Total geoneutrinos, high-Q model	33.7	44.3	48.4	50.9	53.7	68.0
Assumed measurement uncertainty		11 %	6 %	15 %	9 %	4 %

Alternative model of lithosphere

We test two different models that describe the structure of the lithosphere: CRUST1.0[1] (C1) and LITHO1.0[2] (L1). As we already mentioned in Methods section of the paper, C1 describes the crust as $1^\circ \times 1^\circ$ stacks of 6 tiles of a given thickness and uniform density. L1 uses similar vertical layering, but its lateral parameterization is an icosahedron-based tessellation with interpolation between nodes. L1 also gives the structure of the Lithospheric Mantle layer immediately below the Moho, which below continents describes the CLM of laterally varying thickness.

Neither C1 nor L1 provide uncertainties on the thickness of crustal layers or rock density. We expect these to be on the order of 5–10 %. Our geoneutrino flux results reflect only uncertainties in chemical abundances. Magnitude of uncertainty on the crustal structure can be deduced from comparison of results using the two different crustal models. The C1-based model predicts a geoneutrino flux of 50.4 ± 7.7 TNU from the lithosphere at Jinping location (Table 1), while L1-based model prediction is 53.0 ± 7.7 TNU (Table S2). This difference is consistent with L1 Continental Crust around Jinping being thicker compared to CC of C1; globally Continental Crust in L1 is 13% more massive than in C1. The total predicted geoneutrino signal at Jinping in L1-based model is 60.7 ± 7.3 TNU and the mantle (DM+EM) flux is $7.8^{+2.5}_{-2.7}$ TNU, i.e., 13 % of the total.

Table S2. Geoneutrino flux at Jinping predicted with lithospheric structure of LITHO1.0[2].

Reservoir	Geoneutrino flux in TNU		
	Th	U	Th+U
Upper CC + sediments	7.81 ± 0.78	29.43 ± 6.16	37.19 ± 6.95
Middle CC	3.11 ± 0.25	9.14 ± 2.83	12.26 ± 3.08
Lower CC	0.37 ± 0.11	0.91 ± 0.27	1.28 ± 0.38
OC sediments	0.032 ± 0.002	0.100 ± 0.005	0.132 ± 0.007
OC crust	0.012 ± 0.004	0.059 ± 0.018	0.072 ± 0.021
CC + OC	11.33 ± 0.82	39.63 ± 6.78	50.96 ± 7.60
CLM	$0.44^{+0.29}_{-0.17}$	$1.42^{+0.81}_{-0.51}$	$1.86^{+1.09}_{-0.69}$
CC + OC + CLM	11.83 ± 0.86	41.22 ± 6.83	53.04 ± 7.69
Depleted Mantle	$0.67^{+0.16}_{-0.18}$	$3.60^{+0.83}_{-0.97}$	$4.27^{+0.99}_{-1.15}$
Enriched Mantle	$0.81^{+0.42}_{-0.39}$	$2.36^{+2.13}_{-1.44}$	$3.17^{+2.55}_{-1.73}$
DM + EM	$1.53^{+0.42}_{-0.44}$	$6.24^{+2.08}_{-2.21}$	$7.78^{+2.48}_{-2.65}$
TOTAL	13.35 ± 0.88	47.38 ± 6.46	60.74 ± 7.33

References

1. Laske, G., Masters, G., Ma, Z. & Pasyanos, M. Update on CRUST1.0 - A 1-degree global model of Earth's crust. *Geophys. Res. Abstracts* **15**, Abstract EGU2013–2658 (2013). http://igppweb.ucsd.edu/~gabi/crust1.html.

2. Pasyanos, M. E., Masters, T. G., Laske, G. & Ma, Z. LITHO1.0: An updated crust and lithospheric model of the Earth. *J. Geophys. Res.* **119**, 2153–2173 (2014). http://igppweb.ucsd.edu/~gabi/litho1.0.html.

3. Dziewonski, A. M. & Anderson, D. L. Preliminary reference Earth model. *Phys. Earth Planet. Int.* **25**, 297–356 (1981).

4. Rudnick, R. L. & Gao, S. Composition of the continental crust. In Rudnick, R. L. (ed.) *The Crust*, vol. 4 of *Treatise on Geochemistry*, chap. 1, 1–51 (Elsevier, Oxford, 2014), second edn. Editors-in-chief H. D. Holland and K. K. Turekian.

5. Plank, T. The chemical composition of subducting sediments. In Rudnick, R. L. (ed.) *The Crust*, vol. 4 of *Treatise on Geochemistry*, chap. 17, 607–629 (Elsevier, Oxford, 2014), second edn. Editors-in-chief H. D. Holland and K. K. Turekian.

6. White, W. M. & Klein, E. M. Composition of the oceanic crust. In Rudnick, R. L. (ed.) *The Crust*, vol. 4 of *Treatise on Geochemistry*, chap. 13, 457–496 (Elsevier, Oxford, 2014), second edn. Editors-in-chief H. D. Holland and K. K. Turekian.

7. Huang, Y., Chubakov, V., Mantovani, F., Rudnick, R. L. & McDonough, W. F. A reference Earth model for the heat-producing elements and associated geoneutrino flux. *Geochem. Geophys. Geosyst.* **14**, 2003–2029 (2013). arXiv:1301.0365.

8. Arevalo, R., Jr & McDonough, W. F. Chemical variations and regional diversity observed in MORB. *Chem. Geol.* **271**, 70–85 (2010).

9. Arevalo, R., Jr., McDonough, W. F. & Luong, M. The K/U ratio of the silicate Earth: Insights into mantle composition, structure and thermal evolution. *Earth Planet. Sci. Lett.* **278**, 361–369 (2009).

10. Dye, S. T., Huang, Y., Lekić, V., McDonough, W. F. & Šrámek, O. Geo-neutrinos and Earth models. *Phys. Proc.* **61**, 310–318 (2015). 13th International Conference on Topics in Astroparticle and Underground Physics, TAUP 2013; arXiv:1405.0192.

11. McDonough, W. F. The composition of the lower mantle and core. In Terasaki, H. & Fischer, R. A. (eds.) *Deep Earth: Physics and Chemistry of the Lower Mantle and Core*, chap. 12, 145 (Wiley, 2016). American Geophysical Union.

12. Engel, K. L. & McDonough, W. F. Geochemical models of the Earth and the crustal geoneutrino flux. In Ludhova, L. (ed.) *Geo-neutrinos* (Open Academic Press, 2016).

13. Šrámek, O. *et al.* Geophysical and geochemical constraints on geoneutrino fluxes from Earth's mantle. *Earth Planet. Sci. Lett.* **361**, 356–366 (2013). arXiv:1207.0853.

14. Gando, A. *et al.* Reactor on-off antineutrino measurement with KamLAND. *Phys. Rev. D* **88**, 033001 (2013). arXiv:1303.4667.

15. Agostini, M. *et al.* Spectroscopy of geoneutrinos from 2056 days of Borexino data. *Phys. Rev. D* **92**, 031101 (2015). arXiv:1506.04610.

16. Watanabe, H. Geo-neutrino measurement with KamLAND. International Workshop on 04, January 15–16, 2015 in Tokyo (2015). http://www.awa.tohoku.ac.jp/KamGeo/Agenda_files/04_Watanabe.pdf.

17. Han, R. *et al.* Potential of geo-neutrino measurements at JUNO. *Chin. Phys. C* **40**, 033003 (2016). arXiv:1510.01523.

18. Beacom, J. F. *et al.* Letter of Intent: Jinping Neutrino Experiment (2016). arXiv:1602.01733.
